# Predictors of Superior Mesenteric Artery Syndrome: Evidence from a Case-Control Study

**DOI:** 10.7759/cureus.9715

**Published:** 2020-08-13

**Authors:** Nasser A. N. Alzerwi

**Affiliations:** 1 Surgery, Majmaah University, Ministry of Education, Al-Majmaah, SAU

**Keywords:** superior mesenteric artery syndrome, wilkie’s syndrome, aortomesenteric angle, aortomesenteric distance

## Abstract

Introduction

Superior mesenteric artery (SMA) or Wilkie’s syndrome is a rare condition arising due to compression of the third part of the duodenum between the abdominal aorta and the superior mesenteric artery. It is important to explore factors which help in suspicion and early diagnosis of the condition. The aim of this study was to find out if measurements of aortomesenteric angle and distance can predict the occurrence of SMA syndrome. Another objective was to find out if the BMI was correlated with the aortomesenteric angle and distance of the patients.

Methods

A retrospective hospital-based case-control study was conducted in Qimat Rai Gupta Central hospital, Haryana, India from 2018-2020. Out of total 2100 records of acute and chronic abdominal pain patients, only seven cases of Wilkie’s syndrome were confirmed via Contrast-Enhanced Computed Tomography (CECT). Information on age, gender, BMI, duration of symptoms, clinical presentation, aortomesenteric angle, and distance was compared among three groups: Group I-SMA syndrome patients (N=7), Group II- acute abdominal pain patients (N=14) and Group III- chronic abdominal pain patients (N=14).

Results

The hospital prevalence of Wilkie’s was found to be 0.3%. The median age of patients in Group I corresponded to 26 years as opposed to Group II (31.5years) and Group III (30.5 years). There was a statistically significant reduction in the aortomesenteric angle and distance of Group I patients (22º, 6mm) as compared to both Group II (52.5º, 11mm) and Group III patients (52º, 11mm). A moderate correlation of BMI was found with aortomesenteric angle (r=0.479) and distance (r=0.357).

Conclusions

There was a significant reduction in the aortomesenteric angle and distance of the SMA patients as compared to both patients having acute and chronic abdominal pain. The BMI of patients was positively correlated to aortomesenteric angle and distance to the moderate level. Thus BMI along with aortomesenteric angle and distance can predict the presence of SMA syndrome.

## Introduction

The superior mesenteric artery (SMA) syndrome is characterized by acute or chronic upper gastrointestinal tract obstruction due to compression of the third part of the duodenum between the abdominal aorta and the superior mesenteric artery. The estimated incidence of SMA syndrome is 0.1%-0.3%, thereby categorizing it as a rare condition. It is more prevalent among females as compared to males (3:2), with no reported ethnic predisposition. Arterial mesenteric duodenal compression, cast syndrome, or Willkie’s syndrome are the other terms used for SMA syndrome [1, 2].

Pathophysiologically, there is a loss of intervening mesenteric fat pad between the aorta and SMA, which results in a narrower angle between the vessels leading to duodenal compression. The fat pad holds the SMA away from the spine, thus preventing duodenal compression. The normal aortomesenteric angle ranges from 38 to 65º. However, among patients having SMA syndrome, this angle reduces to less than 25º, which in turn reduces the aortomesenteric distance (AMD) to less than 10 mm [1-4].

Important risk factors identified are dietary conditions (anorexia nervosa and malabsorptive diseases), hypermetabolism (trauma and burns), and cachexia causing conditions like AIDS, paraplegia, and cancer. Other risk factors include abdominal aortic aneurysm, congenitally short or hypertrophic ligament of Treitz, duodenal malrotation, Ladd's bands, lumbar hyperlordosis, mesenteric root neoplasm, surgical correction of scoliosis and peritoneal adhesions [1, 2].

The presenting symptoms of SMA syndrome are significant weight loss associated with nausea, vomiting, and recurrent episodes of abdominal pain. The frequency of pain can be intermittent or chronic depending upon the severity of obstruction. But all these symptoms are vague, thus leading to delayed diagnosis when the disease gets progressed to a late stage.

Along with clinical presenting features, various imaging modalities like plain film x-ray, barium x-ray, endoscopy, Doppler ultrasound, computed tomography (CT), and magnetic resonance angiography (MRA) guide the treating physicians to diagnose the condition. CT scan is the modality of choice as the physicians can measure aortomesenteric angle (AMA) and distance, thus confirming the SMA syndrome [5-7].

From the literature review, it’s evident that weight loss is a chief presenting symptom and both aortomesenteric angle and distance get reduced in SMA syndrome as compared to the normal individuals. However, there are a few gaps which need to be filled, like whether the aortomesenteric angle, distance, and BMI can predict the occurrence of SMA or Wilki’s syndrome. Through this study, we tried to evaluate the correlation of aortomesenteric angle and distance with the BMI among both cases of SMA syndrome and individuals not having SMA syndrome. This present study was carried out with the objectives to assess whether the reduction in aortomesenteric angle, aortomesenteric distance and BMI can predict the occurrence of Wilkie’s syndrome and to find out the correlation of aortomesenteric angle and aort mesenteric distance with BMI.

## Materials and methods

This was a retrospective hospital-based case-control study conducted over a period of 2018-2020 in the Department of Gastroenterology of Qimat Rai Gupta Central hospital, Haryana, India from 2018-2020. As superior mesenteric artery syndrome is a rare condition, we decided to include all cases diagnosed to have SMA for the past two years in our study. For this, we reviewed patient records of all cases who underwent esophagogastroduodenoscopies (EGDs) in the previous two years.

We retrieved case records of 2100 patients who had visited the health facility either with acute or chronic pain in the abdomen. Of these, there were 1524 cases of acute abdominal pain and 576 cases of chronic abdominal pain. The superior mesenteric syndrome was suspected among 10 cases, who were advised contrast-enhanced computed tomography (CECT) scan. But only seven patients got the investigations done and were confirmed to have SMA syndrome.

Following this groundwork, we decided to plan and execute a case-control study in the ratio of 1:2. We had seven cases of SMA, so we enrolled 14 cases each of acute abdominal pain and chronic pain in the abdomen as controls, for whom findings of abdominal CECT were available. Thereby, we had three groups with a total sample size of 35 cases.

Group I (Case): It consisted of seven cases of superior mesenteric artery syndrome.

Group II Control): It consisted of randomly selected 14 cases of acute abdominal pain.

Group III (Control): It consisted of 14 cases of chronic abdominal pain selected by random sampling.

The following operational definitions were considered for the purpose of uniformity:

1. Acute abdominal pain: It referred to an episode of sudden onset of severe abdominal pain having a duration of less than 24 hours. There had been such repetitive episodes for the past three months.

2. Chronic abdominal pain: It was defined as a patient presenting with a history of pain for more than three months, which could either be present all the time or maybe recurring in nature.

3. Superior mesenteric artery syndrome: It is a digestive condition occurring due to compression of the duodenum (the first part of the small intestine) between two arteries that is, the aorta and the superior mesenteric artery, leading to a reduction in aortomesenteric angle and distance. The normal value of aortomesenteric angle ranges for 38-65º, which reduces to less than 25º among SMA patients. Similarly, the aortomesenteric distance also gets reduced to 10 mm or below as diagnosed in CECT.

A data extraction sheet was used to record the required information on demographic, clinical, endoscopic, and radiological findings of the selected cases and controls. Demographic information pertained to the age and sex of the patient while the clinical aspect recorded data on the height, weight, clinical onset of symptoms in months, and presenting symptoms of the illness. Endoscopic and CECT findings focussed on reporting aortomesenteric angle and distance of the cases and controls.

Ethical approval was taken from the Institute Ethics Committee prior to commencement of study.

Data were entered and analyzed using SPSS v22.0. As a first step, BMI was calculated from recorded weight and height. Then, descriptive analysis was done to present numerical data of age, duration of symptoms onset, BMI, aortomesenteric angle and distance as their median and interquartile range due to small sample size for all three groups. Gender, a categorical variable, was represented as number and percentage. Mann-Whitney U test was used to compare the median for the above-described variables between two groups, namely Group I vs Group II, Group II vs Group III, and Group I vs Group III. A p-value of less than 0.05 was indicative of a statistically significant difference between the two groups, thus reflecting upon the probable association of independent variable with the outcome. Pearson’s coefficient (r) was calculated to find out the correlation of BMI with aortomesenteric angle and distance for the total sample (N=35).

## Results

Prevalence of superior mesenteric artery syndrome

Over a period of the past two years, we could find 2100 case records who underwent esophagogastroduodenoscopies (EGDS) of which 07 cases were confirmed of having superior mesenteric artery syndrome. Hence, the prevalence of SMA syndrome was found to be 0.3% among the patients coming with either acute or chronic abdominal pain in our hospital setting.

Demographic and clinical characteristics of patients with SMA syndrome and with acute and chronic pain

Clinically the patients of SMA syndrome (Group I, N=7) presented with dysmotility with dyspepsia (n=4), unexplained weight loss (n=2), and reflux with dyspepsia (n=1). Five patients had comorbidities like spina bifida (n=2), anorexia nervosa (n=2), and Crohn’s disease (n=1) as shown in Table 1. The patients in acute abdominal pain (Group II, N=14) presented with the chief complaint of pain in the epigastric region in all cases, whereas chronic abdominal pain (Group III, N=14) patients came with complaint of pain in the epigastric region (n=14) associated with gastroesophageal reflux disease in two patients.

**Table 1:** Demographic, clinical and radiological findings of patients confirmed with superior mesenteric artery syndrome (N=7)

Patient Characteristics	Patient 1	Patient 2	Patient 3	Patient 4	Patient 5	Patient 6	Patient 7
Age	34	40	23	38	26	25	23
Gender	Male	Male	Female	Female	Female	Female	Female
Weight (Kgs)	48.0	57.0	45.0	50.8	50.4	40.5	51.0
Height (m2)	1.59	1.61	1.5	1.52	1.48	1.5	1.54
BMI	19	22	20	22	23	18	21.5
Any co-morbidities reported	No	Yes	Yes	No	Yes	Yes	Yes
Specific co-morbidity		Crohn's disease	Anorexia nervosa		Spina bifida	Anorexia nervosa	Spina bifida
Hospitalization done (Yes/ No)	No	No	No	Yes	Yes	No	No
Clinical Presentation	Reflux with dyspepsia	Dismotility like dyspepsia	Dismotility like dyspepsia	Unexplained weight loss	Dismotility like dyspepsia	Dismotility like dyspepsia	Unexplained weight loss
Time since onset (months)	12	6	4	12	12	8	6
Aortomesenteric angle	15	20	23	15	46	24	22
Aortomesenteric distance	4	6	6	5	6	6	6

The median duration of onset of symptoms was found to be 8 (6-12) months, 2 (1.8-3) months, and 15.5 (11.75-17) months among three groups (Table 2). There was a statistically significant difference in the median duration of symptoms onset between Group I & Group II, Group I & Group III, and Group II & Group III (Table 3).

**Table 2:** Characteristics of patients with Superior mesenteric artery syndrome (Group I), acute abdominal pain (Group II) and Chronic abdominal pain (Group III)

Patient Characteristics	Median (Interquartile Range)			
	SMA patients (N=7)	Acute abdominal pain (N=14)	Chronic abdominal pain (N=14)	Total (N=35)
Age (in years)	26 (23-38)	31.5 (28.8-33.5)	30.5 (28.3-33.3)	31 (26-34)
Weight (in Kgs)	48 (39.4-57)	53.3 (48.7-55.7)	52.4 (48.2-53.9)	52.4 (48.2-54.2)
Height (in mts)	1.52 (1.5-1.6)	1.5 (1.5-1.6)	1.53 (1.5-1.6)	1.54 (1.5-1.6)
BMI (Kg/m2)	20 (18-22)	22 (20.9-23.2)	22 (21-23)	22 (20.5-23)
Aortomesenteric Angle	22 (20-24)	52.5 (43.5-58.5)	52 (43.5-58.5)	50 (38-57)
Aortomesenteric Distance	6 (5-6)	11 (11-12)	11 (10.5-11.5)	11 (10-12)
Duration of onset (in months)	8 (6-12)	2 (1.8-3)	15.5 (11.75-17)	7 (2-15)

**Table 3:** Comparing demographic, clinical onset and radiological findings among three groups

Patient's Characteristics	Group I vs Group II		Group I vs Group III		Group II vs Group III	
	Mann- Whitney U Test value	p value	Mann- Whitney U Test value	p value	Mann- Whitney U Test value	p value
Age (in years)	42	0.601	40.5	0.525	93.5	0.835
Weight (in Kgs)	33	0.233	34	0.263	83.5	0.505
Height (in mts)	44	0.708	45.5	0.793	92	0.782
BMI (Kg/m2)	27.5	0.106	28	0.113	93	0.815
Aortomesenteric Angle	0.5	< 0.001	1	< 0.001	94.5	0.872
Aortomesenteric Distance (mm)	0	< 0.001	0	< 0.001	71	0.291
Duration of onset (in months)	1	< 0.001	11.5	0.005	19	< 0.001

**Figure 1:**
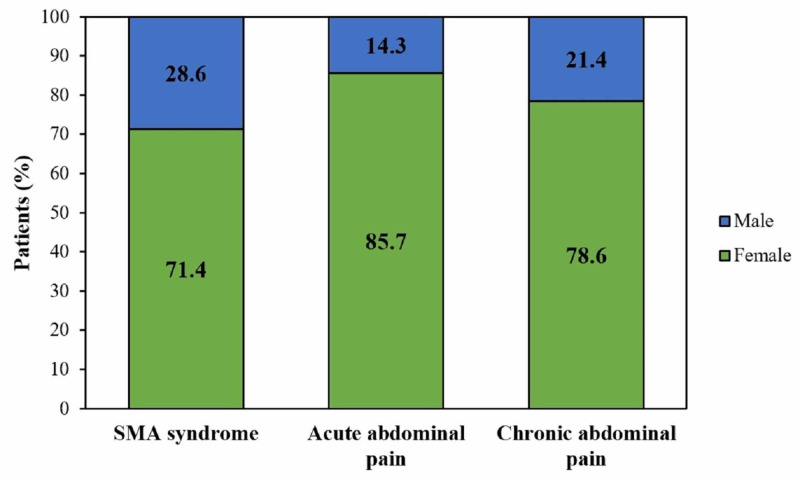
Gender distribution of patients with Superior mesenteric artery syndrome (Group I), acute abdominal pain (Group II) and Chronic abdominal pain (Group III)

The median age of patients with Group I was found to be 26 (23-38) years, whereas the median age in Group II and Group III corresponded to 31.5 (28.8-33.5) and 30.5 (28.3-33.3) years, respectively (Table 2). The proportion of females in the three groups corresponded to 71.4% (n=5), 85.7% (n=12) and 78.6% (n=11), respectively (Figure 1). The median BMI was lower in Group I [20 (18-22) kg/m2] as compared to Group II [22 (20.9-23.2) kg/m2] and Group III [22 (21-23) kg/m2]. However, no significant difference was found for age, gender, and BMI among all the three groups as depicted in Table 3.

Comparing radiological characteristics of patients with SMA syndrome and with acute and chronic pain

The CECT findings revealed that the median aortomesenteric angle in Groups I, II, and III was 22 (20-24) º, 52.5 (43.5-58.5) º, 52 (43.5-58.5) º respectively. Similarly, the aortomesenteric distance was found to be 6 (5-6) mm, 11 (11-12)mm, and 11 (10.5-11.5) mm, in Group I, II, and III (Table 2). As depicted in Table 3, the median aortomesenteric angle and distance were significantly lower in Group I as compared to both Group II (p value<0.001) and Group III (p value<0.001). However, the median aortomesenteric angle and distance were the same in Group II and Group III (p-value 0.872 & 0.29, respectively).

Relationship of BMI with aortomesenteric angle and distance (N=35)

The statistically significant positive correlation of moderate-intensity was found between BMI, and aortomesenteric angle (r=0.479) and aortomesenteric distance (r= 0.357) as represented in Table 4. This implies that if the BMI of a patient decreases, the aortomesenteric angle and distance also reduces.

**Table 4:** Correlation of BMI with aortomesenteric angle and distance (N=35)

Correlation of BMI	Pearson's Coefficient	p value	Interpretation
Aortomesenteric angle	0.479	0.004	Moderate level, Significant
Aortomesenteric distance	0.357	0.03	Moderate level, Significant

## Discussion

The aim of this study was to find out whether factors like BMI, aortomesenteric angle, and distance can predict the occurrence of Wilkie’s syndrome. In other words, we tried to explore if the values of BMI, along with measurements of aortomesenteric angle and distance recorded in Wilkie’s syndrome differ significantly from patients having acute or chronic abdomen. Another objective was to assess if BMI is significantly correlated to aortomesenteric angle and distance. 

For this, we carried out a hospital-based retrospective case-control study by reviewing patient records for the last two years (2018-2019). We found 2100 total cases diagnosed and treated for either acute or chronic abdominal pain. Of these, there were only seven confirmed cases of SMA syndrome. We made three groups for comparison: Group I: SMA syndrome patients (N=7), Group II: Acute abdominal pain patients (N=14), and Group III: Chronic abdominal pain patients (N=14). 

The prevalence of SMA among hospital setting was found to be 0.3% out of the patients who visited the hospital with the acute or chronic abdomen in our study. This is in concordance with the findings of other studies which have also reported the prevalence ranging from 0.1-0.3%, based on radiological investigations [1, 2, 8]. However, a prospective study by Neri et al reported the prevalence of SMA syndrome to be about 0.8% among cases of abdominal pain or dyspepsia [9]. This highlights the view that the prevalence of SMA syndrome is being underestimated due to its presenting symptoms which overlap with many abdominal conditions like ulcers, pancreatitis, eating disorders, etc, causing the disease to go unrecognized for years [10, 11]. A second possible explanation for underestimation of disease burden could be the high responsiveness of patients to conservative treatment, thereby again leading to delay in its precise diagnosis.

The median age of patients in Group I corresponded to 26 years as opposed to Group II (31.5 years) and Group III (30.5 years). All three groups showed a female predilection for the abdominal conditions as shown in Figure 1. The median BMI in Group 1 was found to be lower as compared to Group II and III (Table 2). However, there was no statistically significant difference for these parameters among the three groups (Table 3). The demographics of the SMA patients in our study support the findings of other studies that also reiterate its predominance among young adults (less than 40 years) and females. Sinagra et al also reported no statistically significant difference for age, sex, and BMI among patients with SMA syndrome (Group 1) and patients with any abdominal pathology (Group 2) [8, 9, 12, 13].

CECT findings of aortomesenteric angle and distance were available for the selected patients. There was a statistically significant reduction in the aortomesenteric angle and distance of Group I patients (22º, 6mm) as compared to both Group II (52.5º, 11mm) and Group III patients (52º, 11mm) (Table 2, 3). However, we could not find any statistically significant difference for SMA angle and distance between group II & III. The findings of our study are well supported by the literature, which emphasizes the fact the SMA angle reduces below 25º and SMA distance becomes even less than 8 mm in patients suffering from SMA syndrome [8, 9, 11-15].

For the second objective, we found a statistically significant positive correlation between BMI and aortomesenteric angle and distance (Table 4). This is an important finding as well as a novel aspect of this study. As depicted in table 2, the BMI in Group I was lower as compared to Group II and Group III, though not statistically significant due to the small sample size. Similarly, the aortomesenteric angle and distance in Group I was lower than both the groups. This indicates that as the BMI of a patient decreases, there is the corresponding reduction in SMA angle and distance as reflected by correlation coefficients. Thus, this finding presents a window of opportunity to the gastroenterologist or treating physician for suspecting SMA syndrome. Rather than just depending upon endoscopic findings to guide whether to suspect the presence of SMA syndrome, the reduction of BMI along with the detailed history on risk factors like dieting, or history of scoliosis, etc should ring the bell for the possibility of SMA syndrome. This is to be considered as a strength of this study for generating evidence that BMI should be considered as a factor for screening patients of abdominal pathology for the occurrence of SMA syndrome.

Earlier researchers were unable to report an association between BMI and aortomesenteric angle and distance in a cohort of patients with any abdominal pathology [16-18]. Our study suggests that when BMI of a patient decreases, there is a corresponding reduction in SMA angle and distance, which is the novelty of our study.

Limitations of the study

As this was a case-control study dependent on retrospective patient records of a hospital, it had a few inherent limitations owing to study design. As information on the etiological factors was missing, we could not compare the factors leading to SMA syndrome and other abdominal conditions. Similarly, data on weight loss for the patient was not available which could have been an important indication for suspecting SMA syndrome. Additionally, the small sample size could be considered as another limitation of this study.

## Conclusions

To conclude, we found that the prevalence of SMA syndrome in hospital settings corresponded to 0.3% of the total cases of acute and chronic abdominal pain. There was a significant difference in the aortomesenteric angle and distance of the SMA patients as compared to both patients having acute and chronic abdominal pain. The BMI of patients was positively correlated to aortomesenteric angle and distance to the moderate level.

Future research should focus on conducting adequately powered cohort studies, and that too community-based studies, so that we have a real picture of disease burden. A prospective community-based cohort study would also help to identify different risk factors associated with this disease and would enable the researchers to study the disease progression more comprehensively. We will also be able to study long term patient outcomes in terms of morbidity and mortality, and prognosis of treatment therapies.

## References

[1] Mandarry M, Zhao L, Zhang C, Wei Z (2010). A comprehensive review of superior mesenteric artery syndrome. Eur Surg.

[2] Van Horne N, Jackson JP (2019). Superior mesenteric artery syndrome. https://www.ncbi.nlm.nih.gov/books/NBK482209/.

[3] Lippl F, Hannig C, Weiß W, Allescher H-D, Classen M, Kurjak M (2002). Superior mesenteric artery syndrome: diagnosis and treatment from the gastroenterologist's view. J Gastroenterol.

[4] Ozkurt H, Cenker MM, Bas N, Erturk SM, Basak M (2007). Measurement of the distance and angle between the aorta and superior mesenteric artery: normal values in different BMI categories. SRA.

[5] Bedoya R, Lagman S, Pennington G, Kirdnual A (1986). Clinical and radiological aspects of the superior mesenteric artery syndrome. J Fla Med Assoc.

[6] Fong JKK, Poh ACC, Tan AGS, Taneja R (2014). Imaging findings and clinical features of abdominal vascular compression syndromes. AJR Am J Roentgenol.

[7] Hegde A, Desai S (2009). Superior mesenteric artery syndrome demonstrated by computed tomography. J HK Coll Radiol.

[8] Proaño GAM, Andrade MMC, Rodríguez RAG, Salazar PFG, Aguirre DPC, Poma GVG, Granja BMG (2018). Wilkie’s syndrome, a missed opportunity. J Surg Case Rep.

[9] Neri S, Signorelli S, Mondati E (2005). Ultrasound imaging in diagnosis of superior mesenteric artery syndrome. J Intern Med.

[10] Mathenge N, Osiro S, Rodriguez II, Salib C, Tubbs RS, Loukas M (2014). Superior mesenteric artery syndrome and its associated gastrointestinal implications. Clin Anat.

[11] Welsch T, Büchler MW, Kienle P (2007). Recalling superior mesenteric artery syndrome. Dig Surg.

[12] Rabie ME, Ogunbiyi O, Al Qahtani AS, Taha S, El Hadad A, El Hakeem I (2015). Superior mesenteric artery syndrome: clinical and radiological considerations. Surg Res Prac.

[13] Sinagra E, Raimondo D, Albano D (2018). Superior mesenteric artery syndrome: clinical, endoscopic, and radiological findings. Gastroenterol Res Prac.

[14] Duvie S (1988). Anterior transposition of the third part of the duodenum in the management of chronic duodenal compression by the superior mesenteric artery. Int Surg.

[15] Zaraket V, Deeb L (2015). Wilkie's syndrome or superior mesenteric artery syndrome: fact or fantasy. Case Rep Gastroenterol.

[16] Desai AB, Shah DS, Bhatt CJ, Vaishnav KU, Salvi B (2015). Measurement of the distance and angle between the aorta and superior mesenteric artery on CT scan: values in Indian population in different BMI categories. Ind J Surg.

[17] Lee TH, Lee JS, Jo Y (2012). Superior mesenteric artery syndrome: where do we stand today?. J Gastrointest Surg.

[18] Sahni S, Shiralkar M, Safra Mohamed RC, Jung B, Gaba R, Yazici C (2017). Superior mesenteric artery syndrome: the dark side of weight loss. Cureus.

